# A Holistic Approach for Enhancing Museum Performance and Visitor Experience

**DOI:** 10.3390/s24030966

**Published:** 2024-02-01

**Authors:** Panos I. Philippopoulos, Ioannis C. Drivas, Nikolaos D. Tselikas, Kostas N. Koutrakis, Elena Melidi, Dimitrios Kouis

**Affiliations:** 1 Digital Systems Department, University of the Peloponnese, 23100 Sparta, Greece; p.filippopoulos@uop.gr (P.I.P.); koutrakis@uop.gr (K.N.K.); 2 Information Management Research Lab, Department of Archival, Library and Information Studies, University of West Attica, 12243 Egaleo, Greece; idrivas@uniwa.gr (I.C.D.); dkouis@uniwa.gr (D.K.); 3 Informatics and Telecommunications Department, University of the Peloponnese, 22100 Tripoli, Greece; 4 Museum of Modern Greek Culture, Ministry of Culture, 10555 Athens, Greece; elena.melidi@gmail.com

**Keywords:** museum visitor experience, Bluetooth Low-Energy sensors, indoor proximity tracking, visitor clustering, museum data analytics, ontologies, museum mobile application, museum cultural content management

## Abstract

Managing modern museum content and visitor data analytics to achieve higher levels of visitor experience and overall museum performance is a complex and multidimensional issue involving several scientific aspects, such as exhibits’ metadata management, visitor movement tracking and modelling, location/context-aware content provision, etc. In related prior research, most of the efforts have focused individually on some of these aspects and do not provide holistic approaches enhancing both museum performance and visitor experience. This paper proposes an integrated conceptualisation for improving these two aspects, involving four technological components. First, the adoption and parameterisation of four ontologies for the digital documentation and presentation of exhibits and their conservation methods, spatial management, and evaluation. Second, a tool for capturing visitor movement in near real-time, both anonymously (default) and eponymously (upon visitor consent). Third, a mobile application delivers personalised content to eponymous visitors based on static (e.g., demographic) and dynamic (e.g., visitor movement) data. Lastly, a platform assists museum administrators in managing visitor statistics and evaluating exhibits, collections, and routes based on visitors’ behaviour and interactions. Preliminary results from a pilot implementation of this holistic approach in a multi-space high-traffic museum (MELTOPENLAB project) indicate that a cost-efficient, fully functional solution is feasible, and achieving an optimal trade-off between technical performance and cost efficiency is possible for museum administrators seeking unfragmented approaches that add value to their cultural heritage organisations.

## 1. Introduction

The European culture industry has shown a steady growth trajectory in the last decades, with an annual growth rate that exceeds 10% from 2010 onwards. Based on data from Planeta [1], organisations that are members of the International Council of Museums (ICOM) and maintain and exhibit permanent collections number over 37,000 in 141 countries, and these constitute only part of the culture sector. The total number of museums worldwide sums up to almost 104.000 (Number of museums worldwide by region 2021|Statista, https://www.statista.com/statistics/1201800/number-of-museums-worldwide-by-region/, accessed on 5 January 2024). In Greece, visitors to museums and archaeological sites in 2022 doubled over the previous year (2022) after the expected reduction due to COVID-19, reaching 15.6 million, exceeding the country’s population by more than 50% [2].

Increasing attendance and technological developments are changing how visitors perceive the museum space, exhibits, and collections. Electronic recording and presentation of cultural objects inside the museum space and on the web have helped the transition of interest from the object as a set of material properties to the information surrounding it and how it is presented. An important new aspect of this digital reality is the possibility of documenting and describing to the public the conservation and restoration methods applied to the exhibits. This aspect is of particular interest, both for management and research purposes. It also constitutes a distinct cultural service that deepens and expands the visitor experience [3].

A significant effort has been made in recent years to create and describe related semantic models (e.g., CIDOC Conceptual Reference Model), which will potentially be used to record and encapsulate any cultural information and, thus, facilitate the exchange of data between cultural organisations, research institutes, etc. In addition to broader cultural knowledge, the documentation of conservation work raises important issues related to how this information is recorded. While various data models have been proposed for organising conservation and restoration procedures and methods, more research is required to adopt a common standard and a format for recording the produced knowledge.

Considering the evolution of the concept of culture as a product, cultural heritage institutions are acquiring a new role as providers of education, entertainment, and social interaction services to a heterogeneous audience, thus requiring organisation of the cultural content to meet the needs and demands of diverse groups of visitors [4].

Other concepts of this new digital reality concern how to present cultural artefacts, curate exhibition spaces, understand the impact on visitors (e.g., the time they spend on specific items, the peak hours in a particular area of the museum space) and formulate targeted visitor-centric exhibition strategies and recommendations, in conjunction with visitors’ traffic status and personal preferences. Despite the rapid development and application of techniques to monitor user behaviour within a museum, large-scale data collection and analysis methods have not evolved significantly. Personal questionnaires and observational studies (usually expensive and time-consuming) are still—to this day—widely used as the only reliable source of data for assessing visitor behaviour. Such approaches present significant limitations regarding bias, sample diversity, and size.

From a technological perspective, communication about the documentation of artefacts and conservation processes and the provision of personalised content and navigation to visitors are implemented using information systems that utilise advanced metadata schemas and algorithms to deliver content based on user profiles and behaviour. On the other hand, museum visitor behaviour monitoring falls into a broader area of indoor human detection and tracking, i.e., a demanding and dynamic field of research with special characteristics and numerous applications. An important point of differentiation regarding applicable detection technologies is that, unlike in open spaces, satellite global positioning systems (GPS, Galileo, etc.) cannot be used. To cover indoor environments, a wide range of detection/tracking technologies are used, autonomously or in combination, to achieve the required location accuracy.

The paper presents the MELTOPENLAB (Museum Experience with Location Tracking, Ontologies and Open Laboratories) project (R&D project, co-funded by the EU Regional Development Fund—ERDF, in the context of the Hellenic ESPA Operational Programme, https://meltopenlab.gr/, accessed on 5 January 2024), which addresses holistically the aforementioned issues. MELTOPENLAB focuses on the digital documentation of modern cultural heritage exhibits and their presentation, conservation, and restoration methods. Moreover, the project focuses on developing an advanced information system that captures visitor movement in near real-time and analyses collected data. The system is designed cost effectively, i.e., employing both energy-efficient and widely available technologies, forming a platform for building applications that provide personalised presentation and learning/entertainment experiences to visitors, and tools for evaluating visitor behaviour to museum administrators. 

**Figure 1:**
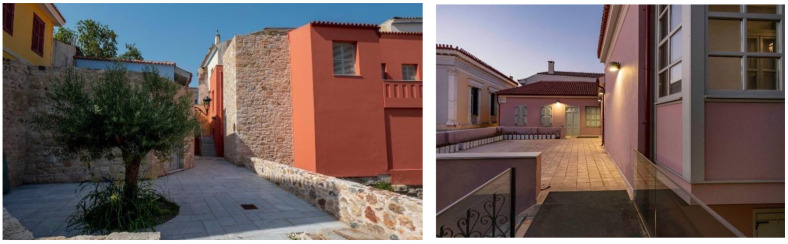
Museum of Modern Greek Culture in Monastiraki, Athens, Greece.

The Museum of Modern Greek Culture (MNEP) (Museum of Modern Greek Culture, in Monastiraki, Athens, Greece. Expected to open to the public in 2024, http://www.mnep.gr/, accessed on 5 January 2024) was selected as a testbed to validate the MELTOPENLAB concept, information system, and applications. MNEP, situated in a multi-building complex in Monastiraki, one of the most touristic spots near the centre of Athens, is the only public museum dealing with the tangible and intangible aspects of modern Greek cultural heritage. For more than 100 years, MNEP has been rescuing, studying, and highlighting everyday life and ritual symbols and objects, information on morals and customs, traditional arts, and techniques that make up the modern Greek reality. Figure 1 depicts indicative views of the MNEP building complex.

The article’s organisation is as follows: Section 2 presents the fundamental technological components the MELTOPENLAB system comprises, and relevant literature, along with rationales for their adoption. The first one is digital content management of the museum exhibits, through traditional metadata schemes and those that enable the implementation of novel services to visitors and curators (i.e., evaluation/reputation ontologies, spatial allocation and navigation ontologies, and conservation/restoration methods ontologies). The second fundamental technological component is visitor detection/tracking. Indoor Positioning System (IPS) topologies, techniques, and technologies are presented and evaluated in the context of the MELTOPENLAB concept. The third component relates to aspects of visitor experience such as visitor movement behavioural models, classification techniques, personalisation, and visitor experience quantification. Section 3 builds on Section 2, presenting the MELTOPENLAB approach in terms of the system’s functional architecture and the rationales for adopting solutions. Section 4 discusses issues related to the implementation and pilot testing of the system, providing preliminary results, and the work concludes with Section 5**,** presenting conclusions and future research.

## 2. MELTOPENLAB Components and Relevant Literature

As the introduction mentions, this section presents the essential technological components comprising the MELTOPENLAB system, along with relevant literature/state of the art, and rationales to help readers understand the choices made within the framework of MELTOPENLAB.

### 2.1. Cultural Content Management

As cultural heritage is continually shaped and transformed in the online world, museums must keep up with two main objectives. First, cross-searching of distributed resources is needed to achieve interoperability of the available cultural content [5]. And second, the integration of data infrastructures and schemas that can be easily used and understood both by museum staff and researchers is another objective [6]. To accomplish these two objectives, a proper metadata structure for curating cultural content is mandatory [7]. A metadata policy is compulsory if a museum seeks to develop a strategic plan for increasing digital access to its collections [8]. Going one step further, according to [9], it is crucial for a proposed metadata schema’s sustainability to be tested in a practical application context, including its description elements and how they “fit and complete effectively” a detailed (if needed) description of an artefact or a collection of artefacts. In related literature reviews, some of the standard metadata schemas for describing museum artefacts and exhibitions are the CIDOR Conceptual Reference Model (CIDOC-CRM), the Visual Resources Association Core (VRA), the Lightweight Information Describing Objects (LIDO) schema, and/or Spectrum. Another metadata schema—and probably the most regularly adopted—is the Dublin Core Metadata Element Set. Around these metadata schemas, a multitude of research efforts have been made. Considering the requirements that stem from the new role being acquired by cultural institutions and the diversification of visitor groups, as discussed in Section 1, different metadata schemas are nowadays required to capture all the issues involved in documenting and presenting artefacts and exhibitions. To address this, MELTOPENLAB considers the following metadata schemas, covering four basic and complementary aspects:

❖ Descriptive Metadata Schemas: The work of Fafalios and colleagues [10] presents the *SeaLiT* metadata schema to describe maritime history information. The authors propose a holistic approach for adopting specific CIDOC-CRM elements to describe artefacts, their semantic relations, and their visualisation through knowledge graphs. In another contribution [11], the authors indicate practical guidelines for proper metadata structure schemas. Among others, the authors highlight the need to describe clearly the purpose of the collection, its specific objectives, the level of richness in descriptions of collections and artefacts, and users and how they will behave when searching for cultural information within the museum repository. Silva and Lara [12] proposed a metadata schema to help museums in the Brazilian region integrate metadata policies to manage and expand access to cultural content properly. The authors’ scope is twofold: first, to suggest combining two metadata schemas (Spectrum and CIDOC-ICOM), and second, to articulate a simple and cost-efficient prototype that museum staff could adopt quickly.
❖ Reputation Metadata Schemas: The metaverse era in the cultural heritage context has flourished. More and more technologies leverage big data, such as virtual museums, photorealistic representations of places, virtual or augmented reality, or application-based navigation tours. For the latter, the visitor downloads and uses the application on a smartphone. The visitor is also asked to provide demographic information and cultural interests, pre-determined by the application developers and museum staff, enabling a personalised experience. Subsequently, the application allows the visitor to rate an artefact or an exhibition in terms of physical presence and how its content is presented (physically and/or within the application). Also, the visitor may rate the whole experience within the museum space and the application itself regarding its usability as a digital vehicle transferring cultural information. Many mobile applications have emerged within the cultural heritage sector, while several general reputation ontologies have been proposed in the business-environment context [13,14,15]. Nevertheless, no reputation metadata schemas exist that allow museum staff to semantically correlate artefacts and collections with high ratings, creating “high rating”-recommended exploration routes in this way.
❖ Preservation Metadata Schemas: Preservation metadata describes “the activities that have been undertaken to facilitate the long-term access to the cultural material” [11]. The purpose here is twofold. First, from the visitors’ perspective, a preservation metadata schema helps to highlight a new viewpoint regarding an artefact, meaning the interventions applied to render the artefact accessible to visitors [16]. Second, from preservation professionals’ and scientists’ perspectives, a preservation metadata schema helps them understand past interventions to materials, allowing them to continue their conservation activities in a well-informed manner. For Velios et al. [17], a preservation metadata schema helps to (i) understand the history of the technology of objects that have been used to restore the material, (ii) identify patterns of damage and specific work trends of conservators, and (iii) evaluate the prior internal environmental management that the artefact has been subjected to. Apart from highlighting the practical benefits of a metadata preservation schema, the author proposes the adoption of the CIDOC-CRM schema to describe the context of the preservation work in detail through its elements. In the MELTOPENLAB context, conservation (semantically encompassing restoration and preservation) metadata is a crucial aspect of its integrated cultural metadata scheme.
❖ Indoor Navigation Metadata Schemas: An aspect that needs to be considered in museum metadata management schemas is the physical location of an artefact or exhibit. From a semantic point of view, establishing an indoor navigation metadata schema helps describe a set of predefined paths/routes within a museum space with the aim that it is utilised by related museum guide applications for enhancing visitors’ experiences and engagement with a cultural entity [18]. One of the first attempts to propose indoor navigation metadata schemas belongs to [19]. The authors proposed OntoNav, a semantic indoor navigation ontology suitable for museum organisations composed of several concepts, such as user, point of interest, passage, exit, corridor, anchor, and path. Another effort related to indoor navigation metadata schemas [20] proposes the iLOC ontology, which is composed of multiple classes and subclasses. Significant classes are the *Location Class*, the *Building Class*, the *Room*, the *Point of Interest* (PoI) and the *Route Section*. In the MELTOPENLAB context, spatial metadata (semantically encompassing the allocation of PoIs and navigation between them) is a crucial aspect of its integrated metadata scheme.

### 2.2. Indoor Detection/Tracking

As discussed in Section 1, the detection/tracking of humans in indoor spaces is a demanding and dynamic field of research, and a wide range of relevant technologies are available today. With the advent of the Internet of Things (IoT) and the rapid development of Machine Type Communication (MTC), there is a great push for applications that take advantage of the end-to-end connectivity now delivered to billions of devices [21,22]. In this context, the focus is on existing and evolving short/medium-range technologies, such as Bluetooth, ZigBee, WiFi, UWB, etc., which belong to Wireless Personal Area Networks (WPANs) and remain integral parts of the IoT “umbrella” [23]. On the other hand, there is a lot of research and commercial development in applications of low-power wide-area (LPWA) technologies [24], such as CSS, SigFox, Lora, DASH7, etc. While providing wide coverage (through private networks and/or mobile cellular networks), the latter do not support high-performance data transfers and do not have the required localisation accuracy in indoor environments. Many authors argue that close cooperation/interoperability between short- and long-range IoT technologies will become necessary to meet different detection/tracking requirements in future networks [25]. 

#### 2.2.1. Topologies and Techniques

Regarding Indoor Positioning Systems (IPSs), there are three topologies/use scenarios that are relevant/have been evaluated in the context of MELTOPENLAB [25,26]:

1. *Device/Mobile Node-Based Localisation—DBL*: The visitor device uses Reference/Anchor Nodes (RNs) to obtain its relative position and provide navigation instructions and is the one that initiates the process and manages location discovery.
2. *Reference Node/Monitor-Based Localisation—MBL*: In contrast to DBL, in MBL the RNs initiate the process, collect the signals from the mobile devices, and transmit them to an appropriate tracking server.
3. *Proximity-Based Detection—PBD*: PBD aims to estimate the proximity (effectively, the distance) between two points of interest rather than their exact location (e.g., coordinates).

In DBL, power consumption, processing, and storage expenses borne by the mobile device are easily manageable in the case of a smartphone, but not in the case of other resource-constrained IoT devices. In MBL, processing and storage are done at the network side (RNs) without incurring resource problems. The main problem is the possible congestion from mass requests to the nodes. PBD is considered a reliable and cost-effective solution for services that use context-aware services, such as advertising, shopping, and logistics. In addition, the related services (Proximity-Based Services—PBSs) are also considered to be energy-efficient solutions and are mainly applied to scenarios based on the movement of people, detecting their presence within a zone.

Given the above, MELTOPENLAB, as it supports the use of wearable e-tickets (resource-constrained devices) for eponymous and anonymous tracking and delivering of context-aware services to visitors, is mainly relevant to Proximity-Based Detection (PBD).

Regarding the basic positioning techniques implemented by the various technologies available today, the following list summarises popular signalling techniques widely used in localisation with a brief evaluation of advantages and disadvantages [25,26]:

❖ RSSI (Received Signal Strength Indicator) [27]: Simple and cost-effective method with no special hardware requirements but low accuracy (in non-LOS cases), noise, and multipath fading issues.
❖ CSI (Channel State Information) [27]: Improved accuracy/robustness to multipath noise over RSSI and more accurate capture of power fluctuations, but no market availability (technology and protocols).
❖ AoA (Angle of Arrival) [28]: Does not require offline measurements and can provide high accuracy, but requires directional antennas and complex equipment/algorithms and exhibits decreasing performance quality with distance.
❖ ToF/ToA (Time of Flight/Arrival) [29,30]: Does not require offline measurements and can provide high accuracy, but requires LOS, strict synchronisation, high bandwidth, and multiple antennas.
❖ TDoA (Time Difference of Arrival) [29,30,31]: Does not require offline measurements and device–reference node (RN) synchronisation. Requires LOS to the receiver, high bandwidth, and synchronisation of at least three RNs.
❖ RToF (Return Time of Flight) [30]: Does not require offline measurements and synchronisation is moderate compared to ToF/ToA. Requires LOS, high bandwidth and is affected by the receiver electronics and delays in an indoor environment.
❖ PoA (Phase of Arrival) [32]: Can be used in conjunction with RSSI, ToA, and TDoA to improve accuracy. It requires LOS to receiver for high accuracy.
❖ FP (Fingerprinting/Scene Analysis) [31,33]: Relatively easy, with many methods (probabilistic, Artificial Neural Networks, k-nearest neighbor, and Support Vector Machine) available for combining off/online measurements. Provides discrete and not continuous location assessment, depending on measurements taken at different times, which change with the environment.

It should be noted that the density of visitor traffic and the complexity of the indoor space seriously impact the ability to maintain the line of sight (LOS) between users and installed equipment (sensors). Techniques potentially applicable in such cases are limited to RSSI, CSI, and Fingerprinting/Scene Analysis. RSSI is cost efficient, but due to signal interference in crowded places, it is difficult to estimate exact locations. Algorithms are employed to reduce these errors to some extent. Such methods are very effective in related IoT services. CSI is more precise but lacks market availability. Fingerprinting may provide high accuracy and reliability but requires increased time and effort to create the fingerprint map, which is also sensitive to environmental changes. Scene Analysis, on the other hand, is less time-consuming and requires less effort, but it is less accurate than Fingerprinting. Given the above, in the case of MELTOPENLAB, where heavy visitor traffic is expected within the complex indoor spaces of MNEP, RSSI seems to be the most appropriate technique, also considering the requirements in terms of cost efficiency and scalability/space re-organisation affecting CSI and Fingerprinting, respectively.

#### 2.2.2. Technologies

The categorisation of detection/tracking technologies [26] depends on the use, or not, of radio frequencies (e.g., Bluetooth, Radio Frequency Identifier, UltraWideBand, etc.), in contrast to technologies that use optical (e.g., LASER, LIDAR), thermal (e.g., IRT Thermal Imaging), or acoustic sensors. In addition, new evolving technologies (e.g., 4D imaging radar), combining intelligent configuration with multiple antennae, and machine learning techniques for data processing/interpretation have been introduced. Finally, there is also a large category based on camera/image processing technologies, which, however, usually require expensive equipment and involve legal issues regarding the use of biometric/personal data.

According to relevant literature [25,26,34], the most common criteria for indoor tracking technologies evaluation include (a) measurement accuracy/variation (depending on many factors, such as noise, LOS, signal propagation, etc.), (b) availability/compatibility (e.g., WiFi and Bluetooth are available in almost all smartphones, which is not the case with UWB or ZigBee), (c) cost of implementation and maintenance (complex factors not limited to equipment), (d) energy efficiency (key parameter for battery-operated devices), (e) latency/delay of response to a location request, (f) scalability in terms of potential to increase (or decrease) the number of nodes, or the geographical size of the system, and (g) system robustness, i.e., the ability to cope with erroneous signals and problems.

MELTOPENLAB has established a series of additional criteria, which are extensions and/or specialisations of the aforementioned general criteria: (a) invasiveness, relating to whether the detection/tracking technology requires for its operation the use of personal information of the user/visitor; (b) dependence on device settings/user options, relating to whether the technology requires user intervention/participation (e.g., activation of device Bluetooth, deactivation of power saving options, etc.); (c) number of users and volume of data produced (e.g., overcrowding may degrade efficiency and accuracy, affecting latency/delay, energy consumption, etc.); and (d) spatial heterogeneity and other characteristics of the covered area (e.g., layout of openings, surfaces of walls, and materials, etc.) affect signal propagation.

Given the above, in the case of MELTOPENLAB, the technology incorporating the most advantages is Bluetooth Low-Energy (BLE), which is also confirmed by the number of existing system implementations in the relevant literature and market. BLE version 5 provides wide coverage, high accuracy (ideally a few tens of cm), and the lowest energy consumption and cost, which is also in line with its high availability in the market (compatible with both Android and iOS). A second choice would be RFID, featuring extremely low-cost passive user tags, that can be used in large numbers inside a closed space, where the limited operation range (approximately 2 m for most commercial solutions) makes it ideal for proximity detection solutions.

### 2.3. Personalised Visitor Experience 

Personalised user experiences in museum environments are mainly provided through recommendation systems, i.e., software systems that maintain the data and correlation between very important parameters in an exhibition/museum, such as (a) content (e.g., supplementary digital material for the exhibits, including multimedia and augmented reality), (b) users (the personal profiles of the visitors, including demographic data and evaluations of exhibits), (c) context (current state of the user and museum environment), and (d) user categorisation/filtering methods (including demographic, content-based, collaborative, social media, hybrid) used for the best possible matching between users and content recommendations. Such parameters are employed to provide recommendations and personalised services [3].

According to relevant literature, measurement/quantification of visitor experiences of a museum/indoor exhibition space is based on statistical data collected and methods of filtering and classification of visitors that are mainly applied during the visit. Various approaches for modelling visitor behaviour, and popular metrics for quantifying visitor experience, based on the aforementioned parameters, are presented in the following paragraphs, including the MELTOPENLAB perspective, where applicable.

#### 2.3.1. Visitor Behavioural Models and Classification Techniques

Various approaches for modelling/classifying visitor behaviour can be found in relevant literature [35]. Some quite popular classifications found in older and recent studies [36] include (a) *ant visitors*, who visit all the points of interest (exhibits) following a linear path; (b) *fish visitors*, who choose mostly the points of interest that are not overcrowded, aiming to obtain a more comprehensive experience; (c) *grasshopper visitors*, with clear choices and interest in specific points; and (d) *butterfly visitors* interested in almost all items; however, the time they spend and their directions of movement tend to vary.

Various methods have been proposed to model visitors as above [35]. The tool described in [37] allows movement analysis and synthesis based on each exhibit’s visit/interaction time and viewing distance. In [38], supervised training of an artificial neural network and unsupervised clustering (classification) method (k-means) testing is described, including parameters such as time spent at each exhibit, number of exhibits visited (compared to the total), and the types of interaction with them.

In [39], the number of exhibits visited, the time visitors spent interacting with those exhibits, and popular visitor paths were used. Popular visitor paths and interaction times were also used in [35] to extract detailed visitor profiles. Group profile extraction seems more difficult and complex, requiring installation of specialised (and thus expensive) equipment within the museum, including gaze detectors. Finally, it is possible to categorise visitor models based on emotion/fitness recognition, providing visitors with wearable smart devices (e.g., wrist- or armbands, smart watches, etc.) [40,41]. Considering cost-efficiency and scalability criteria, group profiling and emotion recognition are outside the MELTOPENLAB context.

#### 2.3.2. Visitor Experience Popular Indicators and Metrics

Statistical indicators and metrics are quite popular for quantifying visitor experience in the relevant literature [42,43], with a focus on the following metrics:

❖ Attraction power (AP): average number of visitors per exhibit.
❖ Revisiting power (RP): average number of revisits per exhibit
❖ Holding power (HP): average and max/min length of visitors’ stays per exhibit.
❖ Heatmaps: visitor density of occupied space per exhibit per period.
❖ Movement trajectories/paths: actual or projected trajectories of visitors, including calculation of popular routes, utilised in visitor traffic management during peak periods, security management of museum spaces, and evaluation of the impact of exhibition strategies.

All the above metrics fall within the MELTOPENLAB scope.

## 3. The MELTOPENLAB System

As already stated in the introduction, MELTOPENLAB addresses holistically and integrates (a) digital documentation of exhibits for presentation and their methods for conservation and restoration, (b) a tool for capturing visitor movement in near real-time (nRT) in both an eponymous and anonymous manner, (c) a mobile application for creating and providing visitors with a personalised presentation, entertainment, and learning experience, and (d) a platform for museum administrators to obtain tracking statistics for the evaluation of exhibits through visitor experience (interest, behaviour, rating). Solution adoption for the above issues and their integration focuses on cost efficiency and scalability, matching the requirements of a multi-space and high-traffic modern museum. In the following paragraphs, the architecture of the MELTOPENLAB system is presented, and crucial aspects of its rationale and implementation are described.

### 3.1. Functional Architecture

**Figure 2:**
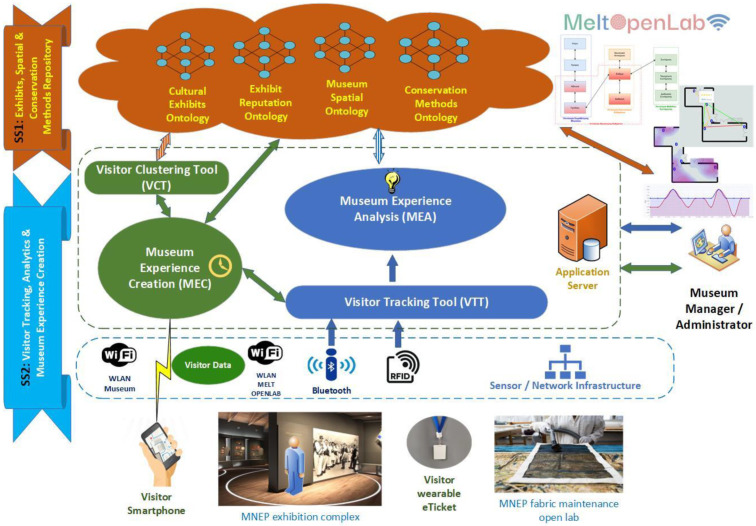
MELTOPENLAB generic functional architecture.

Figure 2 depicts the layers and individual operating components of the MELTOPENLAB generic functional architecture. The overall functionality is divided into two subsystems, one related to the organisation and storage of museum data/metadata, and the other for managing visitor tracking, and feeding location data to a mobile application (visitor front-end) and an application (museum administrators front-end) managing data analytics and visualisation. More specifically, the subsystems can be described as follows:

SubSystem 1—*The exhibits’ description, reputation, and spatial and conservation methods repository*: comprising all four supported metadata schemes, as introduced in Section 1, implemented as an artefacts/data repository (AR). The AR supports four corresponding ontologies, i.e., the Cultural Exhibits Ontology, the Exhibits Reputation Ontology, the Conservation Methods Ontology, and the Museum Spatial Ontology, which are further analysed in the following.

SubSystem 2—*Visitor tracking, data analytics, and museum experience creation*: comprising system tools (VTT, VCT) and end-user applications (MEC, MEA), supported by appropriate sensor/network infrastructure and operated with the use of visitor smartphones and wearable e-tickets (RFID wristbands and BLE neckbands). 

The internal components of the SS2 subsystem are the following:

❖ The *Visitor Tracking Tool (VTT)* is a middleware device that tracks museum visitors, based on Bluetooth Low-Energy (BLE) technology. Arrays of sensors (BLE gateways) were appropriately installed, defining cells corresponding to thematic sets of exhibits, according to museum requirements (e.g., aesthetic issues) and other spatial/technical limitations (e.g., availability of power outlets). Detection of visitors’ presence in a cell was determined by the visitor’s wearable BLE beacon signal strength (RSSI) as received by surrounding BLE gateways, thus implementing a proximity-tracking mechanism that supports near-real-time (updates ranging between 2 and 4 s) detection. A number of additional techniques/methods were applied on a pilot basis to increase accuracy, including (a) equalisation of the signal (linear Kalman filtering used), to smooth fluctuation and minimise noise due to intense multipath fading and shadowing effects, (b) the use of hybrid technology (RFID in part of museum spaces covered) to aid cell detection, using short-range wearable RFID passive tags as e-tickets, (c) spatial filtering employing tables (graphs) of allowed transitions between cells in museum spaces, and (d) use of a Machine Learning Prototype predicting current visitor cell, trained by relevant signal measurements and spatial hints. As depicted in Figure 2, the VTT informs the rest of the SS2 components of the visitor’s current cell. VTT functionality is based on the indoor detection/tracking aspects introduced in Section 2 and further discussed in the sequel.
❖ The *Visitor Clustering Tool (VCT)* enables recommendations and content personalisation for eponymous visitors by providing static (MNEP recommendations)/dynamically created routes comprising selected museum exhibits. The criteria for personalisation are based on visitors’ profiles (static demographic data), their interactions (rating data) determined through the mobile application, and their dynamic movements between cells. VCT functionality is based on the personalisation framework introduced in Section 2 and further discussed in the sequel.
❖ *Museum Experience Creation (MEC)* is a mobile application (developed in both Android and iOS platforms) that acts as a system front-end interface for visitors. MEC functionality includes delivering personalised content if the visitor consents to provide profile data, thus becoming an eponymous user. Anonymous visitors may also use the application to receive generic content relevant to their location in the museum. MEC depends on the VTT for visitor location (current cell), on the VCT for route (static/dynamic) recommendations, and on the system repository (AR) for content regarding cell exhibits and routes. MEC functionality is based on the personalisation framework introduced in Section 2 and further discussed in the sequel.
❖ *Museum Experience Analysis (MEA)* is the front-end application for museum administrators, providing and visualising useful statistics. More specifically, MEA calculates popular indicators such as attraction power (AP), holding power (HP), and revisiting power (RP) by aggregating visitor traffic (eponymous and anonymous) provided by the VTT. The indicators of MEA are stored in the AR, and presented on demand to museum administrators, who may access statistics in various forms (e.g., traffic thermographs–heatmaps, popular routes, user ratings for exhibits, etc.). MEA statistics and visualisations are based on the personalisation framework introduced in Section 2 and further discussed in the sequel.
❖ *Sensor/network infrastructure—visitor e-tickets* are part of SS2 and include the hardware installed in MNEP, covering 16 floors (48 rooms) in nine buildings. The equipment comprises BLE gateways (INGICS iGS01S), wearable beacons (INGICS iBS01), RFID readers/antennae (RodinBell Spider 8000), wearable passive tags (RodinBell W-10), WiFi access points (Ubiquiti Unifi AP AC Lite) and switches/cloud keys collecting BLE and RFID sensor data and relaying it to a central server for processing. To ensure fast implementation in the absence of structured wiring in museum buildings, powerline communication (TP-LINK AV600) adapter pairs were used where applicable.

### 3.2. Artefacts Repository and Ontologies

As mentioned earlier, a crucial component of MELTOPENLAB is the artefacts repository (AR), in which the information on cultural exhibits is organised, mapping all possible relations between artefacts, conservation methods, spatial attributes, and user evaluations, thus defining the following ontologies, based on the framework introduced in Section 2:

❖ The *Cultural Exhibits Ontology (CEO)* combines metadata standards for capturing the artefacts/exhibits descriptive information (metadata schemes used: CIDOC-CRM, VRA, DC).
❖ The *Conservation Methods Ontology (CMO)* aims to provide a standardised way to document methods used for the artefacts/exhibits’ conservation methods (metadata schemes used: CIDOC-CRM, DC).
❖ The *Museum Spatial Ontology (MSO)* represents building and room/floor plans with exhibit allocation and installed sensor coverage areas (cells) (ontology used: iLOC, further addressed in the sequel).
❖ The *Exhibits Reputation Ontology (ERO)* supports the exhibits’ ratings as they are submitted by the visitors and metrics/indicators evaluating their popularity based on visitor behaviour (MELTOPENLAB custom ontology built).

**Figure 3:**
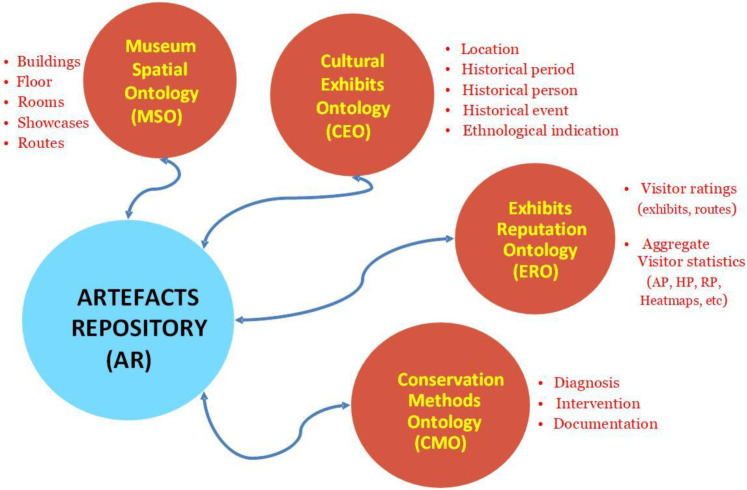
MELTOPENLAB artefacts repository and ontologies.

The proposed ontologies developed in the context of MELTOPENLAB rely on specific ontology engineering principles, defining the purpose of the ontology, and how it contributes to expanding cultural knowledge for museum staff and visitors [44,45]. In the MELTOPENLAB case, ontologies do not work as standalone and static repositories of knowledge; instead, their scope within the AR is to exchange information in a structured and manageable manner with the other MELTOPENLAB components. Figure 3 depicts the four MELTOPENLAB ontologies, their relationships, and basic contents in the AR framework.

**Figure 4:**
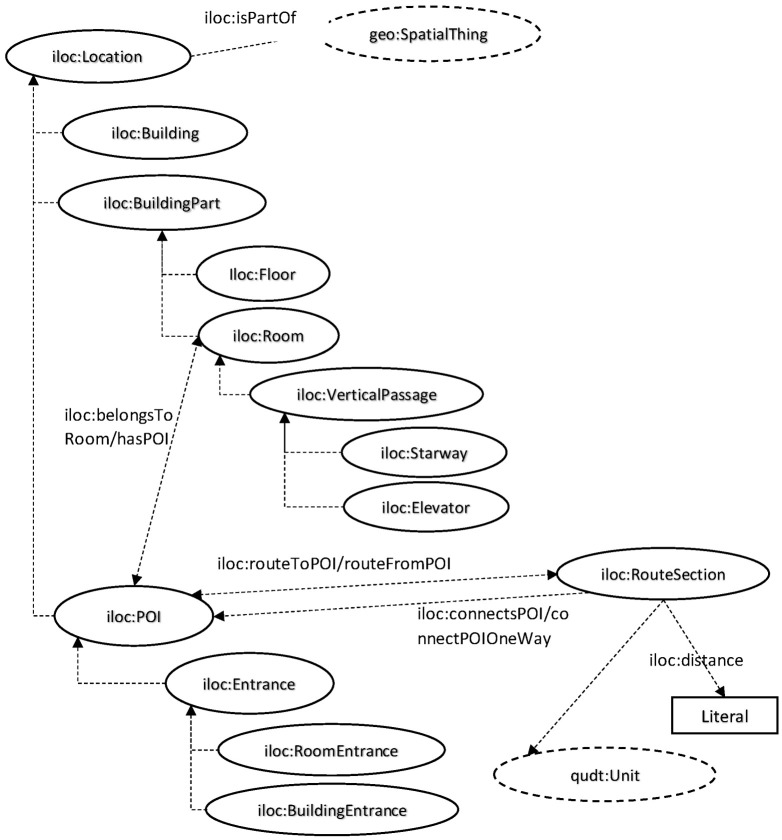
Museum Spatial Ontology (MSO).

To provide an example of an ontology definition in MELTOPENLAB, Figure 4 illustrates (part of) the adopted Museum Spatial Ontology (MSO) based on [20] and the analysis of the involved entities and their interrelations.

The ontology structure (classes and their relations) is the following:

- *Location*: This is used to represent entities that describe locations within indoor spaces and can connect both internal and external areas. It includes subclasses such as Building, Building Part, and Point of Interest (POI).
  ○ Building: Represents the concept of a building, defined as a space with at least one entrance and one exit (which are often the same). In the context of MELTOPNELAB, a Building can be designated as different structures to which an object, exhibit, composite exhibit, collection, or unit can belong.
  ○ Building Part: Provides an abstract concept for different parts of a Building’s internal structure, with subclasses such as Floor and Room.
    ▪ Floor: Gives the concept of a floor or level within a building.
    ▪ Room: Represents the concept of a room, defined as a space with an entrance and an exit (potentially the same). It includes subclasses like Vertical Passage.
    ▪ Vertical Passage: Describes how a user can move to a different floor. This includes subclasses such as Stairway and Elevator.
- *Point of Interest (POI)*: This class describes any point that might interest a visitor, such as an object, exhibit, composite exhibit, collection, or unit. Subclasses include:
  ○ Entrance: This represents all types of entrances and has subclasses such as Room and Building Entrances. These subclasses specifically define connections between rooms or the entry points to buildings.
  ○ Route Section: This represents a path a museum visitor can traverse between two POIs.

The primary relations of the Museum Spatial Ontology (MSO) are the following:

- *Is Part of*: This property expresses a hierarchical structural relationship within the ontology.
- *Connects POIs*: Describes a direct route between two instances of POIs and a sequential series of such direct routes that create a possible navigation between two POIs. In a museum context, this property could be used to develop predefined navigation routes featuring specific objects and exhibits.
- *Connects POIs One Way*: Appears as a parent property of connected POIs to describe one-way routes between Points of Interest.
- *Belongs to Room*: Expresses the relationship between a POI and a Room. A specific Room entity may contain one or more POIs within it.
- *Has POI*: Expresses the inverse property of Belongs to Room. Here, a Room entity can display one or more POI entities.
- *Distance*: Incorporated as a property from the ontology Quantities, Units, Dimensions, and Data Types (QUDTs) to determine the distance in time that a Route Section, which includes a specific number of POIs, covers.

### 3.3. Visitor Tracking Implementation, Personal Devices, and E-tickets

As already mentioned, a decisive factor for determining technical solutions for visitor detection/tracking adopted in MELTOPENLAB was the layout of the museum spaces under consideration and the type of visitor traffic anticipated. 

Proximity-Based Detection (PBD) was the most appropriate tracking method, as the relative position between the visitors and exhibits (distance) and the movements around them was sufficient for estimation in an indoor space, rather than the precise coordinates. The use of wearable e-tickets (as essential visitor tracking equipment, justified in the following) was another reason, as already mentioned in Section 2.2.1.

Signal strength (RSSI) was also indicated in Section 2.2.1 as the prevailing technique, considering dense visitor traffic in MNEP’s complex closed spaces, which make it difficult to maintain line of sight (LOSs) between moving user devices and fixed sensors, as well as cost, market availability, and scalability/exhibition re-organisation requirements.

Bluetooth Low-Energy (BLE) and RFID technologies were also selected (see Section 2.2.2), considering power consumption, cost efficiency, market availability/compatibility, and scalability as critical requirements. The only practical drawback for BLE is its low robustness in an environment with many interferences, which significantly affects the measurements’ accuracy and consistency. Indoor obstacles and noise cause unpredictable signal behaviour and may lead to attenuation degradations, signal distortion, and multipath fading effects, as signals transmitted out of the line of sight (NLOS) make it difficult to estimate the actual distance between moving visitor beacons and fixed receiver sensors.

Such noise factors are difficult to model and are dynamic in nature. Consideration must also be given to the fact that prototypes’ simulations and laboratory test results differ significantly from real conditions, such as those of MNEP, where museum spaces range from small (9–10 m^2^) rooms to large/oblong (60–100 m^2^) halls, with varying openings (windows, doors), surfaces (walls, showcases), and densities of intermediate obstacles. As environmental noise and obstacles cannot be avoided in reality, algorithms are applied to reduce their impact. Median/average, Kalman (KFs), and particle filters (PFs) have been proposed in the relevant literature [26,34] to deal with this issue.

**Figure 5:**
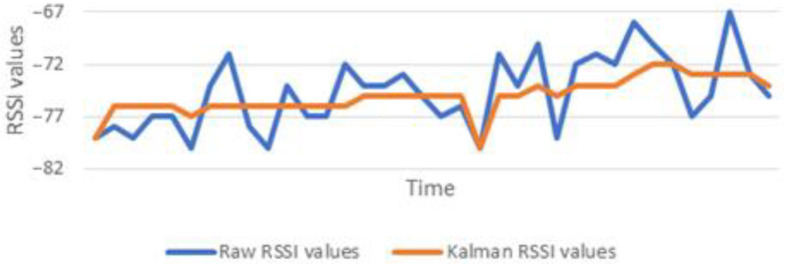
Kalman filter equalisation of BLE RSSIs in MNEP spaces.

Kalman filters, widely used in systems with Gaussian and non-Gaussian noise propagation, were adopted in MELTOPENLAB as an initial attempt to provide an adequate level of signal (RSSI) equalisation regardless of noise. Figure 5 presents an indicative diagram, illustrating preliminary results achieved, smoothing the RSSI fluctuations by a factor of 1/10 after one-dimensional linear KF application.

RFID, being a candidate technology, was finally integrated into the initial design as a hybrid technology to increase cell detection accuracy, rather than as a standalone tracking system. This was mainly due to the size and connection complexity of RFID readers/antennae, a critical factor regarding the aesthetics of the museum’s showcases, limiting RFID installations to only part of MNEP’s spaces. Preliminary results indicate an improvement of about 10% over the raw maximum RSSI cell prediction. 

Graphs of allowed transitions between cells in MNEP spaces (static one-hop tables), were also applied, as an additional layer of spatial/topology filtering/knowledge, built in a deterministic manner, for each museum space to detect and correct possible RSSI-based tracking errors and failures. Preliminary results indicate a maximum improvement of about 3% over the raw maximum RSSI cell prediction.

Finally, a Machine Learning Prototype predicting the current cell was studied (as part of VTT functionality), integrating in its training both pilot signal measurements and hints provided by the above static graphs (e.g., spatially nearest cells), or other knowledge related to movement (e.g., next cell when following a certain route). Training and testing data sets were recorded during project pilots, following a certain mixed movement pattern, involving a max of four users–visitors per cell. Preliminary results indicate a maximum improvement exceeding 20% over the raw maximum RSSI cell prediction (relevant to the specific large museum hall tested, and variations of algorithms used, including K-NN, C4.5, RF, SVM, BayesNet, and Perceptron).

Regarding end-user devices, smartphones cannot effectively and efficiently cover the needs regarding the anonymous tracking of visitors, for several reasons: (a) visitors may not activate Bluetooth, or even use a mobile device during their visit, regardless of the incentives provided by the museum; (b) Android and iOS MAC ID randomisation (https://www.mist.com/documentation/ble-mac-randomization/, last accessed 5 January 2024) make tracking almost impossible, as the Bluetooth MAC address changes frequently; (c) mobile devices’ power-saving schemes result in fluctuations of transmitted power that significantly degrade signal stability, which is required for accurate detection/tracking.

**Figure 6:**
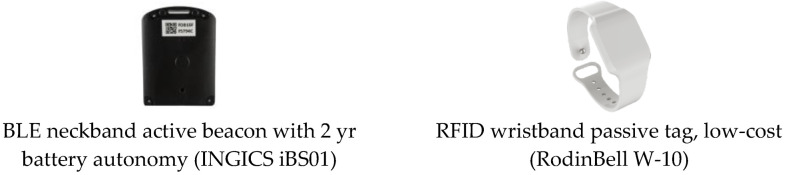
BLE and RFID e-tickets employed in MELTOPENLAB.

Visitor e-ticketing solutions that are popular among museum managers, for security reasons and statistics collection, were adopted in MELTOPENLAB, to address the above user-device issues. Considering low supply and maintenance costs, market availability, and compatibility/interoperability with existing solutions as critical aspects, MELTOPENLAB concluded that the most appropriate e-ticketing technologies to integrate with selected tracking technologies were RFID ARPT (Active Reader Passive Tag) and BLE, in the form of wearable, low-cost wristbands and energy-saving, wearable neckband beacons, respectively, ensuring a stable, detectable signal. Figure 6 depicts the two types of wearable e-tickets supported in MELTOPENLAB.

### 3.4. Museum Experience, Visitor Clustering, and Data Analytics

MELTOPENLAB integrates different functionalities in its architecture, targeting two basic end-user groups (visitors and museum managers/curators). These effectively correspond to two front-end applications: a mobile application (MEC) and the management application (MEA).

The MEC (see Section 3.1) addresses visitors in both eponymous and anonymous modes of operation. Eponymous users, who consent to system detection/tracking and provide the application with profile data (age, educational level, language, and particular preferences), are classified into different visitor categories (six visitor classes supported in the initial project design, relating to the level of understanding/experience and language) and receive content personalised accordingly. 

Visitor classification and content personalisation, including route recommendations, are handled by the VCT functionality, based on static demographic data, visitor interactions (exhibit ratings are aggregated into cells and integrated into route evaluations) and dynamic behaviour (two basic movement models were considered in the initial project design, roughly corresponding to “butterfly” and “grasshopper” visitor models as defined in Section 2.3.1). 

**Figure 7:**
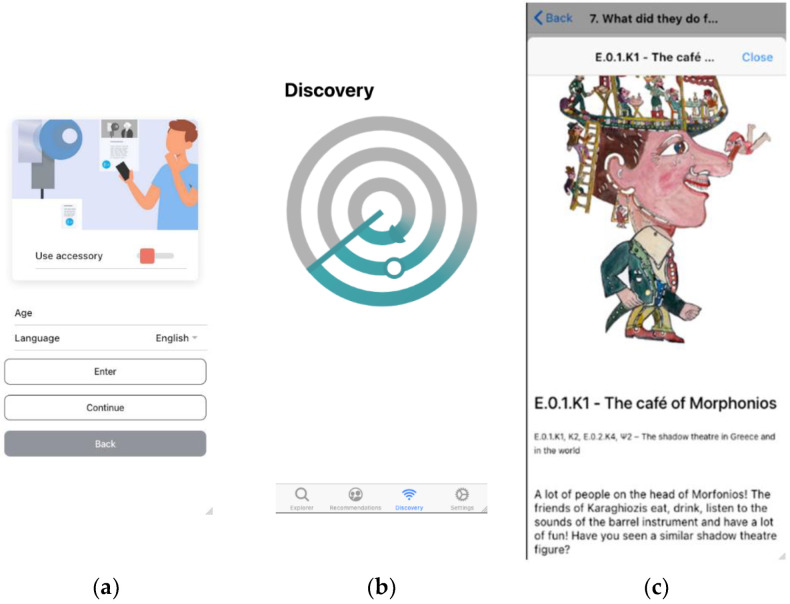
Visitor mobile application (MEC) GUI: (**a**) login page where the user provides personal information and tracking consent; (**b**) tap button triggering visitor location update; (**c**) exhibit presentation content.

Figure 7 depicts indicative screenshots of the MEC application.

**Figure 8:**
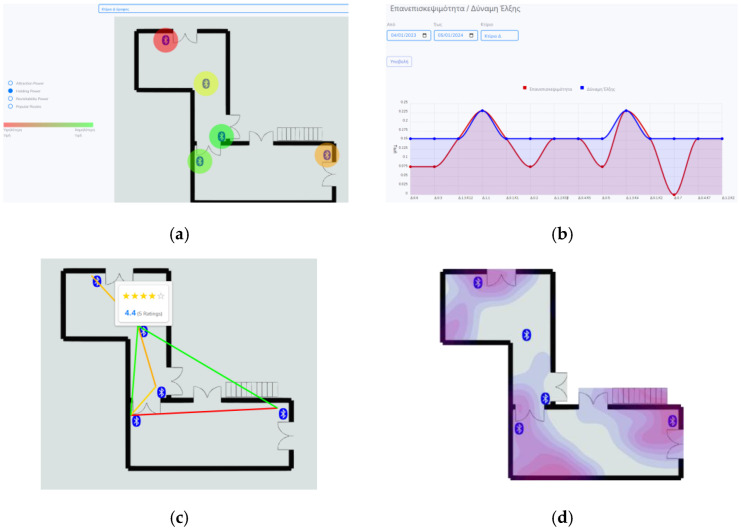
Management application (MEA)—indicative graphs. (**a**) Holding power per cell (red > orange > yellow > green) in museum building D. (**b**) Revisitability (red) and attraction power (blue) per e-ticket and period in museum building D. (**c**) Popular routes (red > orange > green) and visitor ratings. (**d**) Heatmap of museum building D, based on visitor density for a given period.

MEA (see Section 3.1) provides tools for museum administrators and showcase/content curators. More specifically, the MEA application calculates and presents visitor (behaviour) statistics based on recorded traffic data and visualises cell/route evaluations based on visitors’ ratings. MEA aggregates visitor data and processes it asynchronously (offline), regularly feeding (off-loading) statistics/indicators to the repository (AR). Popular statistics (see Section 2.3.2) per cell are supported, including *attraction power*, *revisiting power*, and *holding power*. The presentation encompasses line and bar diagrams, heatmaps (thermographs of visitor densities occupying cell spaces), popular routes between cells and museum rooms, and visitor ratings of exhibits and routes. Figure 8 depicts indicative graphs that the MEA application generates.

## 4. Discussion

The current and following section discuss implementation and evaluation aspects of the MELTOPENLAB approach, including preliminary pilot results, benefits, opportunities, risks, and the practical and managerial implications for museums that adopt similar solutions. MELTOPENLAB future steps are also unfolded.

This paper proposes a holistic approach for enhancing visitors’ experience and overall museum performance, responding to cultural sector research and market gaps, identified in Section 1. Therefore, the primary focus is on integration, rather than seeking excellence per component/technology incorporated. In this context, issues related to the preliminary effort of installing all the technologies required for the whole system to be functional, and subsequently, of operating and maintaining the infrastructure, along with the integration of novel processes ensuring optimal utilisation, become important. From a strategic perspective, museum administrators should be aware that as the proposed solution tends to become more holistic, there is higher staff fatigue resulting from the installation of the plurality of the devices and, subsequently, their becoming familiar with how the system works [46]. Against this backdrop, the management should focus on what the system provides—if it is utilised adequately—and how it contributes to the organisation [47]. Interviews with MNEP administrators and contributing experts, following MELTOPENLAB pilots, indicated that the organisation of targeted demonstration sessions for all staff and specialties involved is required from the initial stages of system installation. This is aimed both at education and the optimisation of the novel processes and tasks introduced. Interviews also indicated that adding value to museum performance, requires time to be assessed, in terms of its impact on both visitors and processes, while there is no standardised methodology for its quantification. 

The adoption and parameterisation of the four ontologies used in MELTOPENLAB, were tested in a practical context to assess their functionalities, interoperability, and utilisation by museum staff involved in exhibit documentation and presentation. An approach has been proposed to assess the potential sustainability of the adopted ontologies [10,11,48]. However, two crucial aspects have been raised and should be considered by museum administrators. 

First, based on the museum content type, there is a need to involve experts who know about the exhibits and collections and can match the available cultural content with the related fields of documentation per ontology. Second, closer expert collaboration is required when more technological integrations are deemed necessary to cover the produced cultural information. In the MELTOPENLAB approach, several experts are involved, including folklorists, historians, conservators, ontology engineers, and sensor and network engineers, etc. This context is aligned with the modern digital humanities ecosystem in which culture, arts, and technologies interplay [48]; nevertheless, museum administrators should pay special attention to pre-organising this grid of multi-versed professionals and scientists with different backgrounds [49,50].

Following the integration perspective, space coverage and complexity, implementation and maintenance costs, and the potential for scalability, including market availability/compatibility, are crucial factors when selecting the most appropriate indoor detection/tracking technology [25,26,34]. The cost factor includes sensors and networking equipment, corresponding consumables (e.g., wearable e-tickets for visitors) and operational and maintenance costs, including future upgrades, both at the hardware and software levels. Based on the assumption that museums struggle with scarce resources, expensive solutions such as visitor gaze detection or visual processing/recognition systems were excluded. The energy-efficient BLE and RFID solutions proposed, offer availability, scalability and flexibility at affordable levels and low maintenance costs, and are considered more sustainable. Initial MELTOPENLAB business modelling scenarios indicated that with an average cost of BLE coverage (including sensors, network, and e-ticket beacons) in the order of EUR 8 per sq. meter, an affordable solution that significantly increases museum performance is not only possible in terms of long-term investment viability, but may also generate significant revenues, considering market penetration and visitor traffic data scenarios for EU-based large museum/cultural organisations.

A relatively low precision in terms of localisation granularity, i.e., localisation occurring at the level of a group/section of exhibits (potentially a small room or hall section), rather than specific objects on display in a showcase, is anticipated with the technologies adopted. In the MNEP case, where exhibits are in principle organised in thematic sections, including large showcases, or series/sets of showcases, proximity zone detection was the actual requirement. However, in other cases, it might be necessary to re-organise exhibits by grouping them into thematic showcases (or series/sets of showcases), or by seeking different system configurations that optimise detection granularity.

A different aspect relating to both scalability and the localisation/detection granularity issues discussed above is the relation between the density of installed sensors and system performance, which is critical for cost efficiency. Considering the relatively small size of the majority of MNEP rooms, the detection/tracking topology adopted (PBD), and the coverage configuration of the BLE system employed, sensor density does not impact performance. More specifically, detectable RSSI thresholds set in the order of -80 dBm define minimum coverage cells (ideally, semicircular disks) in the order of 12 sq. meters. Therefore, the density of the sensors required in an average room (approximately 28 m^2^) for optimal performance is constant. This effectively means that increasing the number of sensors does not improve results; rather, it increases cell overlaps and signal/processing complexity. In this context, system performance/accuracy depends on the layout of the space, as was also verified by preliminary results in processing MNEP’s measurements.

In most systems described in the literature, updating the visitor’s location is considered sufficient if it is near real-time (nRT), that is, without requiring real-time updating in the strict sense. Therefore, the MELTOPENLAB requirement for tracking latency was set accordingly to a range of 2–4 s (VTT cell update), considering that BLE-based systems accumulate delays for processing and denoising of measurements, as well as considering the effects of the anticipated visitor behaviour in terms of average speed when roaming between rooms (47 MNEP halls with areas ranging from 9 m^2^ to 68 m^2^ and one of 105 m^2^), within nine covered buildings, given the specific layout of MNEP. In this context, initial pilot results confirmed the adequacy of this setting in terms of average MEC response time, in the order of 2–5 s. 

MELTOPENLAB pilots regarding system detection accuracy and application performance were carried out in two modes of testing: structured (6 visitors simultaneously present in an average room/section area, with two cells installed and a group of 4 visitors in the cell area, following a pre-determined movement pattern, involving both circular and straight movements at different speeds) and unstructured (larger groups of 8–10 visitors, roaming without pre-determined movement patterns, following, however, static and/or dynamic route recommendations). Structured pilot measurements mainly aimed at recording training/testing data sets used in VTT detection accuracy enhancement using ML algorithms (see Section 3.1), while unstructured measurements focused on MEC application response time and overall user experience (i.e., relevance of content and routes). Prior to application testing, system and component functionality verification and validation, in terms of communication and performance requirements, were successfully conducted. In the sequel, end-user (visitor) and expert (project and museum staff) evaluation of the applications and overall system performance/value was carried out, via the aforementioned structured/unstructured field tests and questionnaires/interviews. The processing of data generated in the pilot testing is ongoing.

## 5. Conclusions and Future Work

MELTOPENLAB addresses holistically and integrates four components: (a) metadata schemas for the digital documentation and presentation of exhibits and their conservation methods, spatial management, and evaluation, (b) a tool for capturing visitor movement in near real-time (nRT) in both eponymous and anonymous mode, (c) a mobile application for creating and providing visitors with a personalised presentation, entertainment, and learning experience, and (d) a platform for museum administrators to obtain tracking statistics for the evaluation of exhibits through visitor experience (interest, behaviour, rating). Solution adoption determination analysis for the above issues and their integration focuses on cost efficiency, availability, compatibility, and scalability, matching the requirements of modern multi-space and high-traffic museums.

Visitor experience is enhanced through the provision of personalised learning/entertainment content and exhibit/route recommendations, dynamically provided, according to visitor profile/preferences and behaviour. Museum managers/curators, on the other hand, obtain access to visitors’ traffic monitoring and exhibit/route evaluation statistics, thus enabling the diversification of cultural content according to the needs of different groups of visitors and the informed redeployment of exhibits’ allocation within museum spaces, according to traffic requirements (e.g., overcrowded spots) and/or periodical focus on specific concepts. They are, thus, enabled to formulate targeted visitor-specific exhibition strategies. Furthermore, the integration of four different ontologies relating to artefacts documentation (CEO), conservation methods (CMO), spatial management (MSO), and exhibit/route evaluation (ERO) expand their ability to record and exchange metadata crucial for their evolving role in the culture sector.

Preliminary results following MELTOPENLAB field pilots in MNEP, a modern multi-space culture museum with high traffic anticipated, indicate that the proposed solution is feasible, achieving an optimal trade-off between technical performance and cost efficiency. However, evaluation/assessment of the pilot results is ongoing, and a number of issues are under study, including the following:

❖ Assessment of the impact of MELTOPENLAB’s added value on visitors and museum management processes, including both internal (e.g., exhibition planning, MNEP staff routines and processes) and external (e.g., ontologies integration, metadata exchanges) aspects.
❖ Sensitivity analysis on the parameters affecting the balance between solution cost efficiency and technical performance (all identified issues, including the amount and range of dynamic data provided).
❖ VTT accuracy improvement using an ML prototype employing variations of different algorithms (see Section 3.1). Preliminary results show a potential to increase the accuracy of cell prediction by a maximum of more than 20% in large halls and about 6% on average, i.e., including small spaces, as compared to the maximum RSSI indication after KF equalisation. Further improvement is expected to be achieved with the use of spatial hints from MNEP’s topology and temporal RSSI features.
❖ VCT functionality may also be enhanced and developed into a recommendation system by applying ML algorithms and utilising combinations of all the aspects of prior visitors’ satisfaction and experience (attraction, revisiting, and holding power).
❖ Combination of sensor-based real-time data with post-visitor experience results (a parallel quantitative survey has been constructed aiming to encapsulate the post-visiting experience), enabling museum staff to achieve optimised monitoring of visitors’ overall satisfaction.

## Data Availability

Data are contained within the article.
